# *Candida auris* skin colonization: mechanisms, microecological interactions, and emerging decolonization strategies

**DOI:** 10.3389/fcimb.2026.1860369

**Published:** 2026-07-30

**Authors:** Mingjing Wei, Yuyuan Xue, Yuhang Mei, Junhao Zhu, Li Li, Xindi Ma, Guo Chen, Min Zhu

**Affiliations:** Department of Dermatology, Huashan Hospital, Fudan University, Shanghai, China

**Keywords:** *Candida auris*, decolonization strategies, immune evasion, metabolic adaptation, skin colonization

## Abstract

*Candida auris*, as an emerging multidrug-resistant pathogen, has become a major threat to global public health due to its high mortality rate, strong transmissibility, and propensity to cause outbreaks in healthcare settings. Persistent skin colonization is a key factor contributing to the high transmissibility of *C. auris* and represents one of its important distinguishing features from other *Candida* species. However, the mechanisms underlying *C. auris* skin colonization remain poorly understood, and relevant prevention and control strategies are still underdeveloped. This article aims to systematically review the epidemiological characteristics of *C. auris* skin colonization, alterations in the skin microbiota, host immunoregulatory mechanisms, colonization mechanisms, and decolonization strategies, thereby providing a theoretical basis for further understanding the pathogenic mechanisms of *C. auris*, optimizing infection prevention and control measures, and developing targeted decolonization intervention strategies.

## 1 Introduction

*Candida auris*, a multidrug-resistant and highly transmissible pathogen, can cause a spectrum of clinical manifestations ranging from asymptomatic colonization to life-threatening bloodstream infections and even central nervous system infections, posing a major threat to global public health (Jeffery-Smith et al., 2018; Jiang et al., 2026). Since the first reported infection in Beijing in 2018 (Wang et al., 2018), *C. auris* infections in China have shown a rapid spreading trend with distinct regional clustering (Tian et al., 2021; Kim et al., 2024; Peng et al., 2024; Miao et al., 2026). Unlike many *Candida* species that are commonly associated with gastrointestinal or mucosal reservoirs, *C. auris* exhibits a pronounced tropism for the skin, enabling it to survive and persistently shed from the human skin surface. Sexton et al., (2021) demonstrated that *C. auris* loads are extremely high in skin sites such as the axilla and groin, and that skin colonization levels are positively correlated with environmental contamination. More recently, strain-resolved metagenomic and isolate-sequencing data from nursing homes showed that resident skin can serve as a reservoir for clonal *C. auris* and other antimicrobial-resistant pathogens, emphasizing the transmission relevance of persistent skin colonization (Proctor et al., 2025). Elucidating the mechanisms underlying *C. auris* skin colonization is of great clinical significance for interrupting its transmission chain and developing precise prevention and control strategies.

The skin microenvironment, comprising pH, microbial communities, temperature, humidity, and stratum corneum integrity, directly influences microbial colonization and survival (Kortekaas Krohn et al., 2024). pH is a key factor regulating the structure of resident skin microbiota and fungal pathogenicity (Pianalto et al., 2024). Although the weakly acidic environment maintained by healthy stratum corneum is not directly fungicidal, it effectively inhibits the growth and proliferation of various opportunistic pathogens, including Candida species (Cottier et al., 2019). The ability of *C. auris* to adapt to the skin microenvironment is a prerequisite for its successful colonization of the skin surface. However, research on how the skin microenvironment influences *C. auris* colonization remains limited. This review systematically summarizes the distribution characteristics of *C. auris* colonization across different skin sites, explores its ecological adaptation mechanisms for epidermal colonization in the context of skin microenvironment regulation, and aims to provide a theoretical basis for clinical infection prevention and control.

## 2 Characteristics of *C. auris* colonization

### 2.1 High-risk populations and clinical features

Epidemiological surveys indicate that *C. auris* colonization predominantly affects males, with a high proportion of elderly individuals aged over 50 years and critically ill patients (Kohlenberg et al., 2022). A comprehensive review on candidemia further emphasizes that skin colonization is not merely a passive reservoir but an independent risk factor for progression to bloodstream infection, with colonization burden showing a positive correlation with infection risk (Dong et al., 2026). These findings underscore the critical need for effective decolonization strategies in high-risk populations to interrupt the transmission chain and prevent invasive disease. *C. auris* colonization is mainly concentrated in long-term hospitalized patients, intensive care unit (ICU) patients, and immunocompromised individuals, particularly those with prior exposure to broad-spectrum antibiotics or azole antifungals (Briano et al., 2022). These clinical associations should be interpreted as a multifactorial context involving healthcare exposure, impaired host defense, altered skin or systemic microbiota, and device-associated surfaces, rather than as the direct biological effect of any single exposure. The ecological imbalance and selective pressure induced by antibiotic exposure create conditions favoring the overgrowth of *C. auris*. Patients using invasive medical devices such as catheters are at significantly increased risk, as these devices not only provide a direct entry route for fungi but also create ideal physical surfaces for biofilm formation (Jones et al., 2024). Comorbidities closely associated with *C. auris* colonization include diabetes mellitus, hypertension, otitis media, malignant tumors, HIV infection, cardiovascular disease, chronic kidney disease, and chronic lung disease (Al-Rashdi et al., 2021). Basic research provides further mechanistic insights into these clinical observations. (Huang et al., (2021) established a mouse skin colonization model using wild-type C57BL/6NTac mice, in which *C. auris* was applied to shaved dorsal skin and ear pinnae and fungal burden was quantified from skin swabs and disaggregated skin tissue, and found that broad-spectrum antibiotic pretreatment, fluconazole exposure, or a high-fat diet to induce diabetes alone did not directly lead to a significant increase in *C. auris* skin colonization load. This suggests that the risk factors observed clinically may not be direct biological consequences of a single factor but may be closely related to high-frequency exposure opportunities in healthcare settings, compromised immune status of patients, and systemic ecological dysbiosis. Thus, the mouse data support, rather than contradict, a clinical model in which persistent colonization emerges from the convergence of healthcare exposure, immune impairment, and microecological dysbiosis.

### 2.2 Skin colonization characteristics and screening strategies

*C. auris* can be recovered from multiple clinical and surveillance specimens, including urine and respiratory samples; however, recovery from these specimens should not be automatically equated with true urinary or respiratory colonization, and *C. auris* also exhibits unique skin affinity and can be found in the external ear canal, wounds, mucosal surfaces, and body fluids (Kohlenberg et al., 2022). Studies have identified the groin as a primary screening site for asymptomatic *C. auris* colonization, particularly in ICU patients, and it has become a core sampling area for active surveillance in clinical practice (Nascimento et al., 2024; Danielsen et al., 2025). This region, characterized by skin folds, high humidity, a relatively neutral pH, and abundant hair follicles, may provide a suitable microenvironment for *C. auris* adhesion, penetration, and biofilm formation (Horton et al., 2020a). Notably, *C. auris* not only remains on the skin surface but can also deeply colonize hair follicles and deeper skin tissues, a property that enables it to establish reservoirs that evade routine surface surveillance, forming an important biological basis for its difficult eradication (Huang et al., 2021). The duration of *C. auris* colonization remains unclear, but patients can remain colonized for extended periods during hospitalization (Caceres et al., 2019). Decolonization of contacts remains challenging because existing screening methods may not identify all colonized sites. One outbreak investigation reported that after three consecutive negative screenings, decolonization of close contacts still resulted in one contact testing positive again, leading to a subsequent strategy of weekly screening until discharge (Schelenz et al., 2016). For colonized individuals discharged into community settings, New York State implemented a pilot case management program, and the results showed that approximately two-thirds of patients colonized during hospitalization did not remain colonized indefinitely after discharge (Bergeron et al., 2021).

## 3 Mechanisms of *C. auris* skin colonization

**Figure 1:**
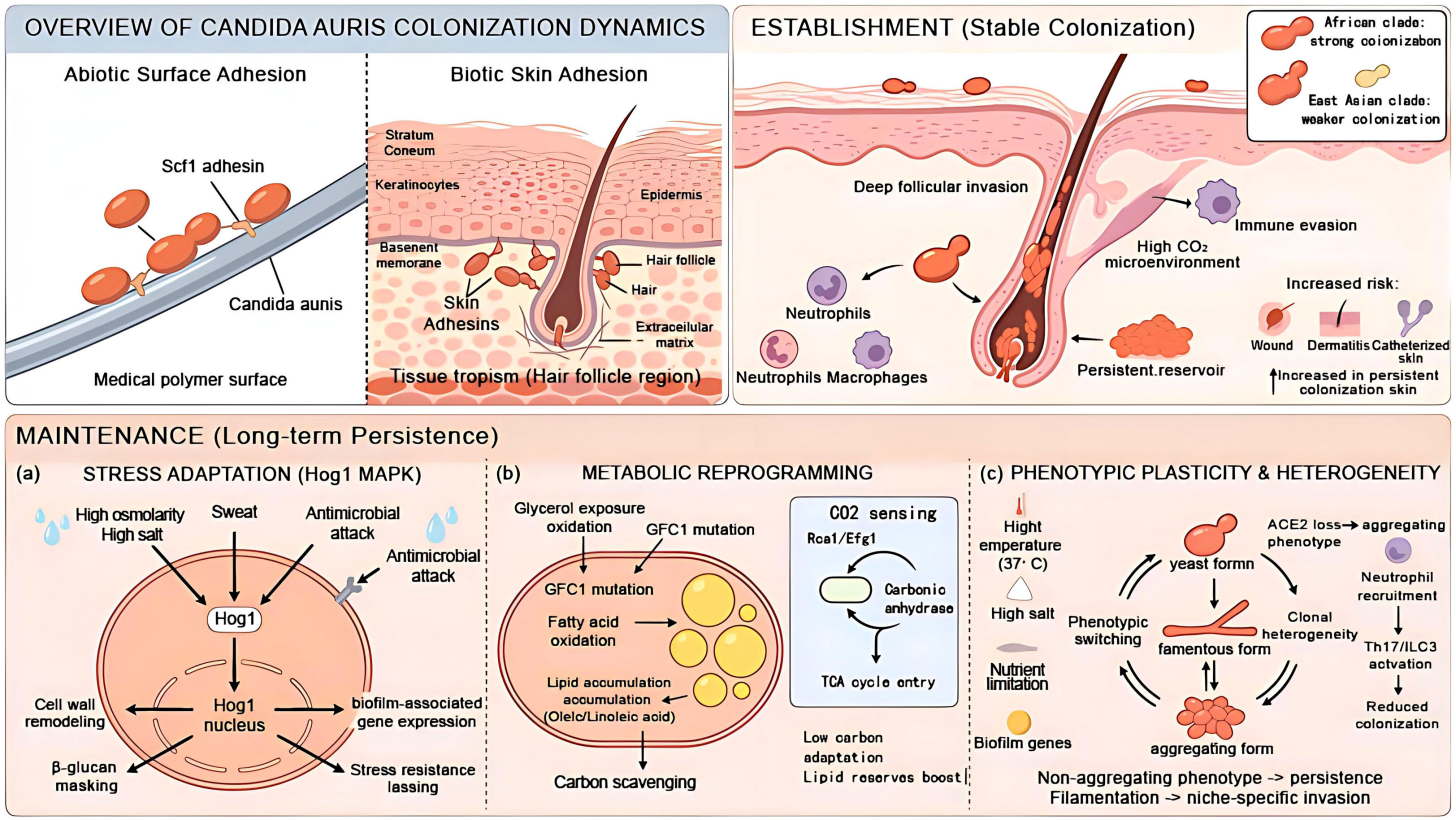
Schematic overview of *C. auris* skin colonization. The process comprises three interconnected phases: initiation, establishment and maintenance. This model highlights the multifactorial strategies underlying *C. auris* long−term skin persistence.

The ability of *C. auris* to persistently colonize human skin relies on a complex, multi−stage process involving initial adhesion, deep tissue invasion, immune evasion, and long−term metabolic adaptation. **Figure 1** provides a schematic overview of these interconnected stages, which are detailed in the following sections.

### 3.1 Initiation of colonization: tissue tropism mediated by adhesins

Efficient colonization of host skin by *C. auris* begins with the recognition of host tissues and medical materials by surface adhesins (**Figure 1**). The adhesin *Scf1* primarily mediates attachment to abiotic surfaces such as plastic and catheters (Santana et al., 2023), while Als family proteins such as *Als4112* confer high tropism for hair follicle areas rich in basement membrane components by binding to keratinocytes and the extracellular matrix (Zhao et al., 2025). Gene knockout experiments showed that *Als4112* deletion reduced *C. auris* binding to laminin by approximately 50%, decreased initial skin colonization capacity in mice by about sevenfold, and significantly improved survival in a systemic infection mode (Zhao et al., 2025). Through this tissue tropism, *C. auris* successfully colonizes the complex skin surface.

### 3.2 Establishment of colonization: deep invasion and immune evasion

After surface adhesion, *C. auris* exhibits a unique skin affinity distinct from *Candida albicans*, enabling it to invade deeply along hair follicles and form biofilm-like structures (Huang et al., 2021). This deep colonization confers significant concealment on *C. auris*; even when skin surface swab tests turn negative, the fungus can survive for months in deeper tissues, serving as a reservoir for sustained nosocomial transmission. In addition to physical protection, the hair follicle niche harbors elevated CO_2_ concentrations due to active epithelial cell metabolism and microbial accumulation. In this high−carbon microenvironment, *C. auris* can directly utilize environmental carbon sources to maintain high levels of fitness (Phan-Canh et al., 2026). Patients with long−term indwelling catheters, dermatitis, or wounds have significantly increased risk of developing persistent colonization due to disrupted skin barrier and direct exposure of deeper tissues (Zhai et al., 2021). Furthermore, Huang et al. (2021) showed that the four major clades differ in murine skin colonization and deeper skin residence, with the African clade showing the highest burden and the South Asian/East Asian clades showing the lowest deeper-tissue recovery (Briano et al., 2022). The mechanisms underlying these clade-level differences remain unresolved. Existing studies identify several skin-fitness determinants that could be examined across clades, including *Scf1*-mediated surface adhesion through exposed cationic residues, *Als4112*-mediated keratinocyte and extracellular-matrix binding, cell-wall mannosylation that modulates neutrophil recognition, and Hog1-dependent stress adaptation; however, direct evidence that any of these factors explains clade-specific deep invasion is still lacking (Horton et al., 2021; Zhai et al., 2021; Santana et al., 2023).

During deep invasion, *C. auris* appears to maintain colonization by combining weak early inflammatory recognition with later immune misdirection. At initial contact, *Als4112*-dependent binding to host cells is not accompanied by canonical NF-κB activation, limiting the early inflammatory signal that would normally promote rapid clearance. Once colonization is established, IL-17R/Act1 signaling provides an important protective axis: in topical murine colonization, impaired IL-17R signaling increases fungal recovery from the skin surface and tissue (Huang et al., 2021). However, *C. auris* can shift the subsequent adaptive response away from this protective pattern. Das et al., (2025) showed that *C. auris* reinfection preferentially induces an IFNγ-dominant CD4+ Th1 response driven by IL-12 from inflammatory macrophages and monocyte-derived dendritic cells; excess IFNγ suppresses IL-17-associated antifungal responses and increases keratinocyte and dermal fibroblast injury, thereby linking immune skewing to barrier damage and fungal persistence. In parallel, single-cell and functional analyses identified an innate evasion mechanism in which *C. auris* induces macrophage-derived IL-1Ra. By antagonizing IL-1R signaling, IL-1Ra reduces neutrophil fungicidal activity; accordingly, recombinant IL-1Ra impaired neutrophil killing ex vivo, whereas IL-1Ra neutralization lowered skin fungal burden *in vivo* (Balakumar et al., 2024b). Together, these findings suggest that *C. auris* persistence is not simply caused by immune suppression, but by a coordinated shift from insufficient early recognition to Th17 and neutrophil dysfunction during chronic colonization.

### 3.3 Maintenance of colonization: metabolic reprogramming and phenotypic plasticity

#### 3.3.1 Metabolic reprogramming and adaptation to extreme environments

Facing stresses in the skin microenvironment, including high osmotic pressure, desiccation, antimicrobial peptides, nutrient limitation, and variable carbon availability, *C. auris* has evolved multi−layered metabolic adaptation strategies. The Hog1 MAPK signaling pathway, a core regulator of environmental stress, helps the fungus withstand the hyperosmolar environment of sweat and phagocyte killing by modulating cell wall composition and biofilm−associated genes (Shivarathri et al., 2024). In simulated sweat, *C. auris* not only survives but also secretes large amounts of extracellular matrix to form dense biofilms; even under extreme conditions where water evaporation concentrates salt by 2.5−fold, its biofilms remain viable for over 14 days (Horton et al., 2020b). This inherent tolerance closely matches its preferential colonization of warm, sweat−prone areas such as the axilla and groin (Casadevall et al., 2019).

Regarding nutrient acquisition, remodeling of lipid and carbon metabolism is central to maintaining colonization. Host-derived nutrient availability is niche-specific: moist sites such as the axilla and groin provide sweat-derived salts, urea, lactate, amino acids, and microbial metabolites; sebaceous areas provide lipid-rich substrates; and dry keratinized sites are relatively nutrient-poor and desiccating. The daily chemical glycerol can induce mutations in *GFC1*, relieving inhibition of fatty acid β−oxidation and leading to a 30%–50% accumulation of intracellular oleic and linoleic acids, providing the necessary lipid metabolic foundation for skin colonization (Deng et al., 2024).Under extreme nutrient stress, *C. auris* can also form large lipid droplets to store triglycerides and sterol esters, a physiological state that confers significant tolerance to skin antimicrobial peptides (e.g., LL−37) and enhances survival on desiccated medical fabrics (Zheng et al., 2025). Recent work under physiologically relevant skin-like conditions further suggests that *C. auris* can exploit low-abundance sweat- and sebum-associated nutrients and that fatty-acid oxidation and glyoxylate-cycle genes, including *FOX2*, *CAT2*, and *ICL1*, support adaptation to these niches (Nicklas et al., 2026). More critically, in response to the carbon−deficient environment of the skin surface, *C. auris* relies on the carbonate−sensing pathway (CSP) regulated by transcription factors Rca1 and Efg1. This pathway efficiently captures trace CO_2_ by upregulating carbonic anhydrase Nce103, converting it into bicarbonate that feeds into the tricarboxylic acid cycle to provide basal energy for initial colonization; knockout of this pathway dramatically reduces skin colonization burden (Phan-Canh et al., 2026).

#### 3.3.2 Heterogeneity and phenotypic plasticity

Beyond underlying metabolic adaptations, *C. auris* exhibits remarkable phenotypic plasticity, switching among yeast, filamentous, aggregating, and White-Brown states, and even forming phenotypically diverse heterogeneous populations within clonal lineages. Recent evidence identifies White-Brown switching as a high-frequency program influenced by temperature and carbon source and involving regulators such as Wor1, Msn4, Crz2, Rca1, and Efg1; this switching affects adhesin expression, stress tolerance, antifungal susceptibility, immune evasion, host adaptation, and fitness on murine skin (Phan-Canh et al., 2025). Importantly, the contribution of distinct morphologies to colonization is context-dependent rather than uniformly beneficial. In specific lipid-rich settings, *GFC1* mutation or exogenous fatty acids can induce filamentous growth that may enhance tissue invasion (Deng et al., 2024), and earlier systemic-infection studies indicate that aggregative or filamentation-competent states may influence virulence outside the skin niche (Chen et al., 2018; Bing et al., 2024). However, current skin-infection data do not support the simple conclusion that aggregation promotes persistent high-burden skin colonization. In an *ACE2* deletion model, aggregative *C. auris* strongly induced neutrophil recruitment as well as Th17 and type 3 innate lymphoid cell activation, and these immune responses were accompanied by significantly lower skin fungal burden than that observed with non-aggregative wild-type or ACE2-reintegrated strains (Balakumar et al., 2024a). Therefore, persistent high-burden skin colonization appears to favor a non-aggregating, immune-evasive yeast-like state, whereas aggregation can increase immune visibility and reduce skin persistence.

### 3.4 Comparison with *Candida albicans*: species-specific cell wall and biofilm features

*C. albicans* tissue invasion is strongly linked to yeast-to-hypha transition and hypha-associated adhesins, whereas *C. auris* usually shows limited true hyphal formation under standard laboratory conditions yet colonizes skin efficiently. This suggests that *C. auris* relies more heavily on skin-tuned surface adhesion, immune-evasive cell wall architecture, stress tolerance, and biofilm matrix protection. *C. auris* biofilm matrix polysaccharides can sequester triazole antifungals and contribute to drug tolerance (Dominguez et al., 2019), and its cell wall mannosylation contributes to neutrophil evasion through pathways divergent from *C. albicans* and *C. glabrata* (Zhao et al., 2025). Solid-state NMR further shows that *C. albicans* and *C. auris* differ in their adaptive cell wall responses to echinocandins (Dickwella Widanage et al., 2025).

### 3.5 Translational limitations of current experimental models

Most mechanistic evidence on adhesins, immune evasion, metabolism, biofilms, and deep skin invasion derives from murine models, ex vivo skin systems, or *in vitro* assays. These models are essential but should not be overinterpreted. Murine skin differs from human skin. Native human skin better preserves the epidermal barrier but is limited by donor variability and ex vivo conditions, whereas porcine skin is useful for topical-delivery testing but does not fully reproduce chronically colonized patients with disrupted barriers, polymicrobial communities, medical devices, and prior antifungal or antiseptic exposure (Seiser et al., 2024).

## 4 Interactions between *Candida auris* and the skin microbiota

Human skin is colonized by diverse commensal microorganisms that help defend against pathogen invasion by secreting antimicrobial molecules and modulating local immunity (Hsu and Yassin, 2025).However, the specific role of the skin microbiota in *C. auris* colonization remains unclear. Proctor et al., (2021) monitored 10 skin sites in 57 nursing home residents and first revealed the dynamic relationship between *C. auris* colonization and skin microbiota structure. The study categorized fungal communities into four states: CST1 (*Malassezia*−dominant, healthy homeostasis), CST2 (mixed Candida species, transitional), CST3 (predominantly *Malassezia slooffiae*), and CST4 (*C. auris*−dominant, colonized state). The transition rate from CST2 to CST4 was as high as 38%, whereas CST1 rarely transitioned directly to CST4, suggesting that colonization requires a transitional phase involving multiple Candida species. Once CST4 is established, colonization can persist for over a year, consistent with clinical observations of persistent colonization and recurrent re−positivity. The 2025 Nature study from the same group extends this concept by demonstrating clonal *C. auris* and ESKAPE pathogens on the skin of nursing-home residents, thereby establishing persistent skin colonization as a clinically relevant transmission reservoir (Rossoni et al., 2020).

Bacterial communities also undergo significant changes. The skin of colonized individuals was enriched in Proteobacteria (e.g., *Proteus mirabilis*, *Klebsiella pneumoniae*), whereas negative samples were dominated by commensals such as Staphylococcus. This shift—reduced commensals and increased opportunistic bacteria—creates a favorable niche for *C. auris*. Mechanistic studies revealed that Proteobacteria carry urease, which decomposes sweat urea to release ammonia and CO_2_, and *C. auris* utilizes this CO_2_ as a carbon source through its carbonate−sensing pathway, forming a metabolic commensalism that promotes its colonization (Phan-Canh et al., 2026).

Clinical data support the value of microbiota restoration. After successful decolonization, the proportion of CST4 decreased from 16.0% to 0%, CST1 increased from 68.0% to 79.3%, fungal diversity increased, and Proteobacteria decreased. The dynamic evolution from healthy homeostasis through a mixed transitional state to a colonized state provides targets for clinical intervention. Protecting commensal microbiota and inhibiting intermediate Candida species may represent new prevention and control strategies.

Regarding active intervention, probiotics such as Lactobacillus species show anti−colonization potential through competitive exclusion. Live Lactobacillus paracasei and its postbiotics inhibit *C. auris* growth, disrupt biofilms, and reduce persister cell viability (Mulet Bayona et al., 2022). The mechanisms include organic acid release to lower pH, bacteriocin secretion, and interference with quorum sensing. Such interventions promote epithelial repair and reduce the risk of chemical disinfectant−induced resistance. Therefore, sequential introduction of probiotics after aggressive clearance may help maintain microecological homeostasis and prevent recolonization.

## 5 Decolonization of *Candida auris* from the skin

### 5.1 Colonization detection methods and their limitations

Current *C. auris* colonization screening primarily relies on culture methods, which offer simplicity and low cost but have limitations including long turnaround times and limited sensitivity, leading to false−negative results. Traditional phenotypic and biochemical identification methods are prone to misidentification because of the close phylogenetic relationship between *C. auris* and other *Candida* species (Deng and de Hoog, 2026; Gold et al., 2026). MALDI-TOF MS has become a clinically valuable identification method when the reference database contains validated *C. auris* spectra; recent U.S. hospital data indicate that MALDI-TOF MS is now the predominant yeast-identification method in many acute-care and long-term acute-care settings (Sasoni et al., 2022). However, outdated libraries or unusual spectra can still cause misidentification, so suspicious isolates require confirmation with updated MALDI-TOF databases, sequencing, or species-specific molecular assays. Chromogenic media serve as an important complement to culture methods. The novel chromogenic medium CHROMagar Candida Plus offers the advantages of simplicity and intuitive interpretation, with *C. auris* colonies appearing light blue with a blue halo, achieving 100% sensitivity and specificity after 36 hours of incubation (Franco et al., 2024). However, a limitation is that some *Candida parapsilosis* strains exhibit similar morphology on this medium, and suspicious isolates still require complete identification by mass spectrometry or molecular methods (White et al., 2021). Molecular methods (PCR/qPCR) offer high speed and sensitivity, particularly suitable for screening *C. auris* colonization specimens, but their limitation is susceptibility to interference from residual fungal DNA when assessing decolonization efficacy (Wong et al., 2025). For example, after chlorhexidine treatment, culture methods show a decrease in viable counts, but PCR Ct values do not change markedly, suggesting that residual fungal DNA can lead to discordance between molecular results and actual clinical colonization status (Seiser et al., 2024). Molecular identification can be achieved by sequencing the ITS region of ribosomal DNA, offering both high sensitivity and species specificity, but requires specialized equipment and expertise (Gussin et al., 2025).

### 5.2 Current decolonization methods and clinical efficacy

Commonly used decolonization methods include chlorhexidine, povidone−iodine, and ultraviolet irradiation, but no universally accepted standard protocol currently exists. Chlorhexidine is the most widely studied first−line option; however, its fungicidal effect when used alone is limited. Recent clinical shedding data also suggest that chlorhexidine bathing should be considered a risk-reduction measure rather than a guaranteed eradication strategy (Moore et al., 2017). Moore et al (Rutala et al., 2019) found that chlorhexidine requires combination with alcohol to exert effective killing. Proctor et al (Hsu and Yassin, 2025) demonstrated that chlorhexidine effectiveness is influenced by multiple factors including skin site, cleaning procedures, and microenvironment; while killing susceptible commensals, it selects for naturally tolerant Gram−negative bacteria, giving them a competitive advantage, and this microbiota remodeling may create conditions favoring *C. auris* colonization. Infection control strategies must balance the broad−spectrum killing effect of disinfectants with the maintenance of skin microecological balance. Povidone−iodine is recommended by Public Health England for skin preparation before invasive procedures, but studies report variable efficacy, which is highly dependent on contact time (Cadnum et al., 2018). Regarding physical methods, far−UVC radiation with a peak wavelength of 233 nm can kill 99.9% of *C. auris* at skin−tolerable doses, independent of resistance profile (Fayed, 2025).

## 6 Future decolonization strategies for *Candida auris*

**Figure 2:**
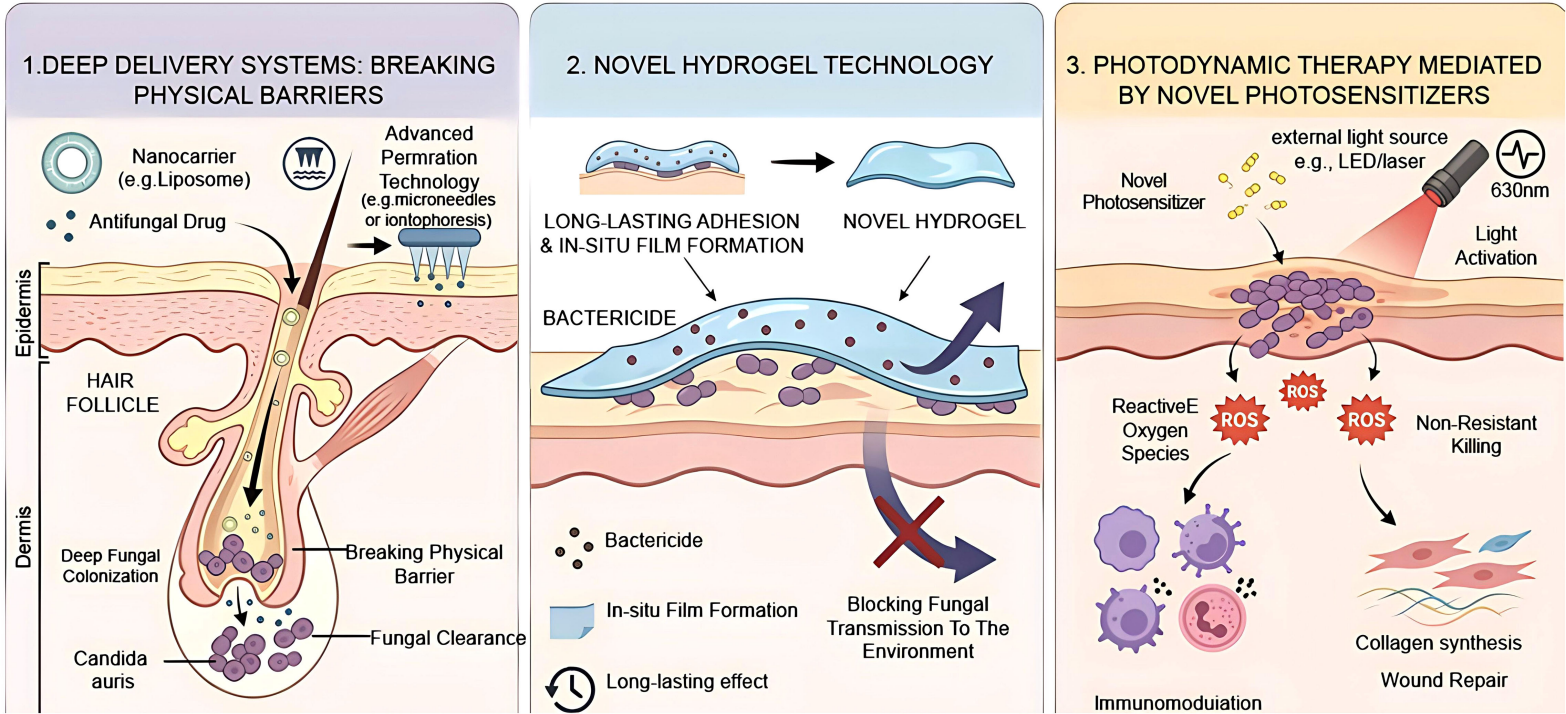
Overview of emerging *C. auris* decolonization strategies. (1) Nanocarriers or microneedles deliver antifungals into hair follicles and deep colonization sites. (2) Hydrogels provide sustained drug release and form a physical barrier to reduce environmental shedding. (3) Photodynamic therapy generates reactive oxygen species for resistance-independent fungal killing. These approaches remain investigational and require clinical validation.

Emerging strategies designed to overcome deep follicular reservoirs, prolong topical activity, reduce environmental shedding, and provide resistance-independent fungal killing are summarized in **Figure 2**.

### 6.1 Deep delivery systems to overcome physical barriers

The sequestration of *C. auris* deep within hair follicles is a core physical barrier to successful decolonization. Nanoparticles (NPs), owing to their small size and modifiable surface properties, can significantly enhance the penetration of antimicrobial molecules (Hassan et al., 2025). Nanocarriers substantially improve the penetration and safety of conventional drugs or natural active molecules; formulating plant−derived molecules such as carvacrol and tea tree oil into nanoemulsions not only effectively disrupts fungal cell membranes but also greatly reduces skin irritation (Jaromin et al., 2026). Jaromin et al (Buonanno et al., 2024) designed cationic liposomes encapsulating the novel antifungal compound PQA−Az−13, which significantly reduced biofilm burden in ex vivo tissues. Liposomal formulations of sphaeropsidin A have also been shown to effectively inhibit biofilm formation and reduce fungal adhesion to epithelial cells (Cleare et al., 2020). Additionally, Cleare et al. (2020) developed a nano−delivery system that, after penetrating biofilms, continuously releases free nitric oxide (NO), eradicating deep viable fungi through irreversible oxidative stress. Targeting the deep niche of hair follicles, the advanced penetration technology (APT) developed by Elshaer and colleagues (Elshaer et al., 2023) uses macromolecular penetration−enhancing excipients to encapsulate chlorhexidine, successfully overcoming the penetration limits of conventional water−soluble disinfectants, providing the most direct evidence for developing novel topical decolonization formulations targeting hair follicles. These approaches remain largely preclinical and should be evaluated against clinically realistic barriers, including hair follicles, disrupted skin, polymicrobial communities, and prior antifungal or antiseptic exposure.

### 6.2 Novel hydrogel technologies

Novel hydrogel technologies address the challenges of prolonged retention of topical agents and prevention of environmental transmission. To extend retention time, Bostănaru−Iliescu and colleagues (Bostănaru-Iliescu et al., 2025) developed a chitosan−based hydrogel loaded with nystatin and propolis. Its intrinsic cationic properties enable strong tissue adhesion and direct electrostatic disruption of negatively charged fungal cell membranes, reducing viable counts on skin models below the detection limit within 6 hours. More promising for clinical translation is *in situ* film−forming hydrogel (Haidari et al., 2020), which rapidly crosslinks upon skin contact to form a semi−occlusive, transparent elastic film. By releasing nano−silver (AgNPs) that penetrate over 80% of biofilms, it provides a sustained moist antimicrobial microenvironment. The physical barrier formed effectively seals colonized sites, preventing desquamation containing fungal elements from shedding into the ward environment due to friction with clothing, thereby physically interrupting the source of contact transmission.

### 6.3 Novel photosensitizer−mediated antimicrobial photodynamic therapy

For complex colonized infections involving skin disruption, chronic wounds, or catheter insertion sites, conventional whole−body bathing often proves inadequate. In such cases, antimicrobial photodynamic therapy (aPDT), which combines physical killing with biological modulation, offers a novel approach. Liu et al (Liu et al., 2026) first reported a PDT strategy based on novel cage−like hypocrellin derivatives. Unlike chemical approaches relying on antibiotics, this strategy generates bursts of reactive oxygen species (ROS) upon irradiation at specific wavelengths, directly causing lethal oxidative damage independent of target−site mutation−based resistance mechanisms. More importantly, while efficiently clearing fungi from wounds, this therapy also effectively modulates local host immune responses, promoting wound healing. Mechanistic work further suggests that caged-hypocrellin-mediated PDT can induce chromatin remodeling and disrupt mitochondrial energy metabolism in multidrug-resistant *C. auris* (Liu et al., 2025). This approach, which does not induce resistance and simultaneously promotes tissue repair, represents an excellent option for managing extremely challenging local colonization by pandrug−resistant strains.

### 6.4 Synthetic antimicrobial peptides and cell-wall-targeted compounds

Synthetic or engineered antimicrobial peptides represent another exploratory antifungal direction. Recent reviews highlight their broad-spectrum activity, rapid membrane- or cell-wall-targeting mechanisms, and potential for rational engineering against WHO-priority fungi, including *C. auris* (Roque-Borda et al., 2025) Cell-wall-targeted peptide-like compounds, such as urease-derived peptides, and lipopeptaibiotics such as coniontins have also shown experimental activity against Candida species or multidrug-resistant *C. auris* by impairing cell wall integrity or sensitizing fungi to existing antifungals (Chen et al., 2025; Perin et al., 2025). These approaches remain preclinical; peptide stability, cytotoxicity, delivery, cost, and *in vivo* efficacy must be resolved before they can be considered for skin decolonization.

## 7 Discussion

The high mortality and outbreak frequency of *C. auris* are closely linked to its unique skin affinity and persistent colonization (Jeffery-Smith et al., 2018; Wang et al., 2018; Jiang et al., 2026). This review systematically examines the initiation of epidermal colonization, mechanisms of deep invasion, microecological interactions, and the evolution of decolonization strategies. Collectively, these findings indicate that *C. auris* skin colonization is not a superficial event but a complex ecological adaptation involving anatomical features, host immunity, and metabolic commensalism.

Mouse models have confirmed that *C. auris* can deeply invade hair follicles owing to its high tropism for basement membrane components (Al-Rashdi et al., 2021). Simultaneously, it avoids early inflammation through *Als4112*-mediated adhesion and escapes Th17-mediated clearance by maintaining a non-aggregating phenotype (Balakumar et al., 2024a; Zhao et al., 2025). C*. auris* also exploits specific mutations to enhance invasiveness in lipid-rich environments and utilizes the carbonate-sensing pathway (CSP) to capture trace CO_2_ for survival on the carbon-deficient epidermis (Deng et al., 2024; Phan-Canh et al., 2026).

Skin microecological dysbiosis plays a synergistic role in *C. auris* colonization. The four-state model (CST1–CST4) proposed by Proctor et al. describes the progressive transition from a healthy Malassezia-dominated homeostasis to a C. auris-dominated state (CST4) (Hsu and Yassin, 2025). Routine clinical use of broad-spectrum disinfectants such as chlorhexidine, while effective in the short term, causes non-specific microecological disruption and selects for urease-carrying Proteobacteria (Gussin et al., 2025). These bacteria decompose sweat urea to release ammonia and CO_2_, providing critical metabolic substrates for *C. auris* through the CSP pathway (Phan-Canh et al., 2026). This cross-kingdom interaction reveals that intensified disinfection can create a vicious cycle of dysbiosis and pathogen recolonization. Future infection control should shift from merely pursuing potent sterilization toward an integrated model that balances pathogen clearance with maintenance of microecological homeostasis.

Decolonization strategies are undergoing a paradigm shift driven by the convergence of materials science and microecology. Novel delivery systems address the physical barrier of hair follicles: Elshaer et al. used advanced penetration technology to encapsulate chlorhexidine, overcoming penetration limits in ex vivo porcine skin (Cleare et al., 2020). Chitosan-based hydrogels provide sustained release and tissue adhesion (Elshaer et al., 2023), while *in situ* film-forming hydrogels create a semi-occlusive physical barrier that seals deep reservoirs and reduces environmental shedding (Bostănaru-Iliescu et al., 2025). Antimicrobial photodynamic therapy offers resistance-independent killing and promotes tissue repair, showing promise for localized lesions associated with chronic wounds or catheter sites (Haidari et al., 2020). Far-UVC and probiotic interventions are also transitioning from proof-of-concept to clinical translation (Rutala et al., 2019).

Several challenges remain. First, most mechanistic and delivery data are derived from ex vivo or *in vitro* models (Huang et al., 2021; Phan-Canh et al., 2026); large-scale randomized controlled trials are urgently needed. Second, murine data indicate that major geographical clades differ in skin colonization and deep tissue residence, but the causal mechanisms—including adhesin regulation, cell-wall remodeling, immune recognition, and stress adaptation—remain unresolved and require systematic comparative testing (Horton et al., 2021; Huang et al., 2021; Santana et al., 2023; Zhao et al., 2025). Third, future strategies should elucidate key nodes of cross-kingdom interactions, such as inhibiting urease activity of accompanying Proteobacteria or precisely blocking the CSP pathway (Phan-Canh et al., 2026), alongside developing rapid point-of-care diagnostics to guide decolonization timing and endpoint determination.

Finally, environmental contamination plays a critical role in transmission (Miao et al., 2026). An ideal decolonization regimen should integrate nano-enhanced or photodynamic targeted killing to overcome physical barriers (Cadnum et al., 2018; Buonanno et al., 2024; Fayed, 2025; Hassan et al., 2025; Jaromin et al., 2026), film-forming materials to seal desquamated skin (Elshaer et al., 2023; Bostănaru-Iliescu et al., 2025), and microecological interventions to remodel the skin niche (Proctor et al., 2021). By constructing a closed-loop system encompassing the host, microbiota, and environment, it may ultimately be possible to interrupt the nosocomial transmission chain and eliminate this major public health threat.
